# Ultrastable Sodium–Sulfur Batteries without Polysulfides Formation Using Slit Ultramicropore Carbon Carrier

**DOI:** 10.1002/advs.201903246

**Published:** 2020-04-22

**Authors:** Qiubo Guo, Shuang Li, Xuejun Liu, Haochen Lu, Xiaoqing Chang, Hongshen Zhang, Xiaohui Zhu, Qiuying Xia, Chenglin Yan, Hui Xia

**Affiliations:** ^1^ School of Materials Science and Engineering Nanjing University of Science and Technology Nanjing 210094 China; ^2^ Herbert Gleiter Institute of Nanoscience Nanjing University of Science and Technology Nanjing 210094 China; ^3^ Soochow Institute for Energy and Materials Innovations College of Energy Key Laboratory of Advanced Carbon Materials and Wearable Energy Technologies of Jiangsu Province Soochow University Suzhou 215006 China

**Keywords:** insoluble polysulfides, low self‐discharge, room‐temperature sodium–sulfur batteries, ultramicropores, ultramicroporous coffee carbon

## Abstract

The formation of the soluble polysulfides (Na_2_S*_n_*, 4 ≤ *n* ≤ 8) causes poor cycling performance for room temperature sodium–sulfur (RT Na–S) batteries. Moreover, the formation of insoluble polysulfides (Na_2_S*_n_*, 2 ≤ *n* < 4) can slow down the reaction kinetics and terminate the discharge reaction before it reaches the final product. In this work, coffee residue derived activated ultramicroporous coffee carbon (ACC) material loading with small sulfur molecules (S_2–4_) as cathode material for RT Na–S batteries is reported. The first principle calculations indicate the space confinement of the slit ultramicropores can effectively suppress the formation of polysulfides (Na_2_S*_n_*, 2 ≤ *n* ≤ 8). Combining with in situ UV/vis spectroscopy measurements, one‐step reaction RT Na–S batteries with Na_2_S as the only and final discharge product without polysulfides formation are demonstrated. As a result, the ultramicroporous carbon loaded with 40 wt% sulfur delivers a high reversible specific capacity of 1492 mAh g^−1^ at 0.1 C (1 C = 1675 mA g^−1^). When cycled at 1 C rate, the carbon–sulfur composite electrode exhibits almost no capacity fading after 2000 cycles with 100% coulombic efficiency, revealing excellent cycling stability and reversibility. The superb cycling stability and rate performance demonstrate ultramicropore confinement can be an effective strategy to develop high performance cathode for RT Na–S batteries.

## Graphical abstract

The coffee residue derived ultramicroporous carbon material loading with small sulfur molecules (S_2–4_) as cathode material for room temperature (RT) Na–S batteries can effectively suppress the formation of polysulfides (Na_2_S*_n_*, 2 ≤ *n* ≤ 8). Due to the special reaction mechanism, the ultramicroporous carbon loaded with 40 wt% sulfur (ACC‐40S) exhibits good rate capability, low‐self discharge, and excellent cycling stability.

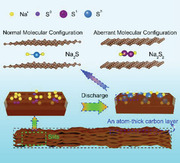

## 1 Introduction

To integrate the renewable energy sources such as wind and solar energies into large‐scale grids, it requires to develop highly efficient energy storage technologies with high energy density, long cycle life, and low cost.^[^ ^1^ ^]^ Among various energy storage devices, metal–sulfur batteries, such as Li–S and Na–S batteries, are especially attractive owing to their high theoretical energy densities and the high natural abundance of sulfur.^[^ ^2^, ^3^ ^]^ However, the scarcity of lithium resource leads to the increasing cost of lithium metal and makes Li–S batteries less attractive for grid‐related applications. In comparison, the abundant sodium resource on earth spur the strong interest to explore Na–S batteries as efficient energy storage technology for large scale grid application. Traditional Na–S batteries are usually operated beyond 300 °C with molten electrodes and solid *β*‐alumina (NaAl_11_O_17_) electrolyte, which induces severe safety concerns and additional costs, limiting their large scale applications.^[^ ^4^ ^]^ Therefore, room temperature (RT) Na–S batteries using liquid electrolyte attract great attention in recent years as low‐cost energy storage systems for large‐scale grid application.^[^ ^5^ ^]^ The major challenge for RT Na–S batteries is the nonconductive nature of S, which causes the low reactivity of S with Na and incomplete reaction to form polysulfides (Na_2_S*_n_*, 2 ≤ *n* ≤ 8).^[^ ^6^ ^]^ The polysulfides dissolution and shuttling in the liquid electrolyte result in fast capacity fading,^[^ ^7^ ^]^ low coulombic efficiency, and large self‐discharge, restricting the practical application of RT Na–S batteries.^[^ ^8^ ^]^

As of now, sulfur loading of 70 wt% and above is viewed as the norm for Li–S batteries because Li–S batteries have been well studied in recent years and people now are working hard for practical applications. Na–S batteries, however, are still at the beginning stage of research now and the sulfur loadings in recent works on Na–S batteries are in the range of 40–60 wt%.^[^ ^9^ ^]^ As the solubility of sodium polysulfides is even higher than that of lithium polysulfides, finding an effective way to suppress sodium polysulfides formation is currently the top challenge for Na–S batteries. To improve the electrical conductivity and suppress polysulfides dissolution, various S host materials, such as carbon, conducting polymer, and metal oxides/sulfides, have been developed with composite cathode designs.^[^ ^6^, ^10^, ^11^ ^]^ Although significant progress has been achieved, the intrinsic problem of polysulfides formation cannot be completely ruled out and the electrochemical performance still cannot meet the requirements for large‐scale RT Na–S batteries.^[^ ^11^ ^]^ Among the carbon hosts, the design of microporous carbon was demonstrated to be very effective to improve the electrochemical activity of S and enhance the cyclability due to the micropore confinement. Two function mechanisms of micropore confinement have been proposed in previous works. By using molecular structure simulation with a cylinder pore model, Xin et al.^[^ ^10^ ^]^ believed that only Na_2_S_2_ and Na_2_S can be formed in the micropores due to the geometry confinement and small sulfur molecules (S_2–4_) in the microporous carbon. Wei et al.^[^ ^3^ ^]^ also found that only Na_2_S_2_ and Na_2_S can be formed in the microporous carbon, however, they attributed this behavior to a quasi‐solid‐state reaction because they believed that metal ions need to desolvate to enter the micropores. Nevertheless, previous works on microporous carbon indicate that the formation of soluble polysulfides can be effectively suppressed while the formation of insoluble polysulfides such as Na_2_S_2_ still exists for RT Na–S batteries. The insoluble polysulfides, as intermediate reaction products, have poor electronic conductivity and could slow down the reaction kinetics and impede the complete reaction to the final product of Na_2_S, thus reducing the reaction efficiency and specific capacity. Therefore, a one‐step reaction with Na_2_S as the only product and no polysulfide formation could be the ideal electrode design for RT Na–S batteries, which could simultaneously attain large specific capacity, good rate performance, and long cycle life. Such highly efficient electrode materials for RT Na–S batteries, however, have not been developed by far.

Herein, we report a micropore confinement cathode that enables one‐step reaction without formation of polysulfides for RT Na–S batteries. The microporous carbon with a major micropore size around 0.5 nm and high surface area of 1100 m^2^ g^−1^ was prepared from the coffee residue. By using density function theory (DFT) calculations, it is indicated that only small sulfur molecules (S_2–4_) can stay in the slit ultramicropores less than 0.6 nm. After infusing the sulfur into the micropores of the carbon, the carbon–sulfur composite electrode with 40 wt% sulfur loading (ACC‐40S) exhibited only one voltage plateau for both charge and discharge and further in situ UV/vis spectroscopy measurements demonstrated Na_2_S is the only product during the discharge. The DFT calculations indicate that Na_2_S_2_ molecules cannot keep its corrugated configuration in the micropores less than 0.7 nm due to the space confinement effect, while the Na_2_S molecules with the planar structure can be formed without distortion in micropores less than 0.5 nm. By using the ACC‐40S as cathode, the RT Na–S batteries exhibited a high specific reversible capacity of 1492 mAh g^−1^ at 0.1 C and excellent cycling stability with nearly no capacity fading for 2000 cycles at 1 C. Importantly, the RT Na–S batteries using the ACC‐40S cathode also exhibited low self‐discharge with a low capacity‐fading rate of 0.17% per day, making them promising for potential applications in large‐scale energy storage.

## 2 Results and Discussion

**Figure 1:**
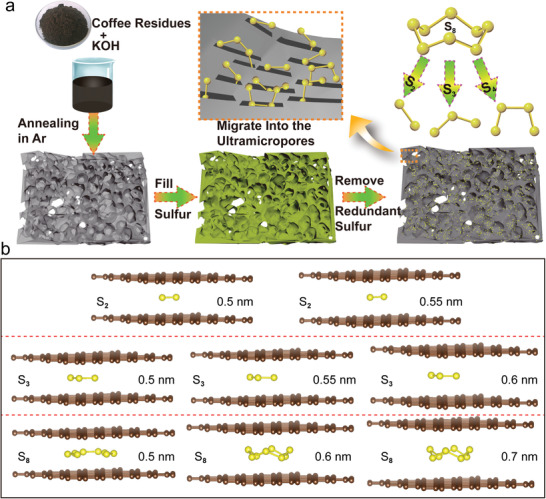
a) Schematic illustration of the synthesis procedure for the microporous carbon and sulfur composite with small sulfur molecules confined in the carbon micropores. b) Optimized molecular structures of S_2_, S_3_, and S_8_ confined into the bilayer graphene with different interlayer spacings.

The facile preparation of ACC and the process of immobilizing sulfur into the carbon ultramicropores are schematically illustrated in **Figure 1**a. ACC can be easily obtained by activating the coffee residues in KOH and annealing in Ar atmosphere. To prepare the carbon–sulfur composite, the commercialized sulfur powder was infused into the ACC material through a traditional melting‐diffusion method. During this process, the initial low temperature treatment makes the sulfur infiltrate into the mesopores and macrovoids of carbon evenly, then the high temperature treatment causes S_8_ molecules to break into the metastable S_2–4_ small molecules and migrate into the ultramicropores.^[^ ^12^ ^]^ The space‐confinement effect of ultramicropores not only limits the volatilization of S_2–4_ at high temperatures, but also inhibits their recovery to S_8_. To understand why only small sulfur molecules can exist in the ultramicropores, the adsorption energies of S_2–5_ and S_8_ molecules inside ultramicropores were calculated by using a bilayer graphene model to simulate the slit ultramicropore. The optimized structures of S_2–5_ and S_8_ molecules inside the bilayer graphene with different interlayer spacings are shown in Figure 1b and Figure S1 (Supporting Information). When the interlayer spacing is decreased to 0.5 nm, only small sulfur molecules such as S_2–4_ could stably exist inside the bilayer graphene, and the buckled S_5_ and S_8_ molecule structures will finally break into S_2_ and S_3_. The adsorption energies of the various S‐molecules inside the bilayer graphene are summarized in Table S1 (Supporting Information). The negative energy means the insertion of an S molecule is an exothermal reaction, which is thermodynamically feasible. It is found that the insertion of S_5–8_ molecule into the bilayer graphene can reach a stable state only when the interlayer spacing is larger than 0.7 nm. In contrast, the more positive of the adsorption energy indicates the smaller possibility of the existence of S‐molecules. Therefore, if the micropore size of the microporous carbon can be controlled less than 0.7 nm, only small S_2–4_ molecules can stably exist in the slit micropores.

By controlling the sulfur infiltration, the carbon–sulfur composites with various sulfur loadings of 40, 50, and 60 wt% were successfully obtained and denoted as ACC‐40S, ACC‐50S, and ACC‐60S, respectively. Figure S2a,b (Supporting Information) show the photo images of the ACC‐40S sample before and after heating. It is obvious that the color changes from gray to black after heating, which suggests the infiltration of sulfur into carbon. Figure S3a (Supporting Information) shows the field emission scanning electron microscopy (FESEM) image of the pristine carbonized coffee residue (CC) without any further treatment, revealing a relatively dense structure without obvious pores being observed. With KOH activation, the obtained ACC sample as shown in Figure S3b (Supporting Information) presents a 3D porous structure with microsized pores interconnnected. In this 3D porous structure, the surface area of the ACC can be greatly increased, and most of the micropores in the carbon can be easily accessed by the small sulfur molecules. Figure S3c (Supporting Information) shows the FESEM image of the final obtained ACC‐40S sample. The well preserved 3D structure and the smooth surface indicate all of the sulfur is infused into the micropores of the carbon without extra sulfur on the carbon surface. The Figure S4a,b (Supporting Information) show the FESEM images of the ACC‐50S and ACC‐60S samples, respectively, which reveal rough carbon surface, suggesting extra sulfur on the carbon surface when the sulfur content is exceeding 40 wt%.

**Figure 2:**
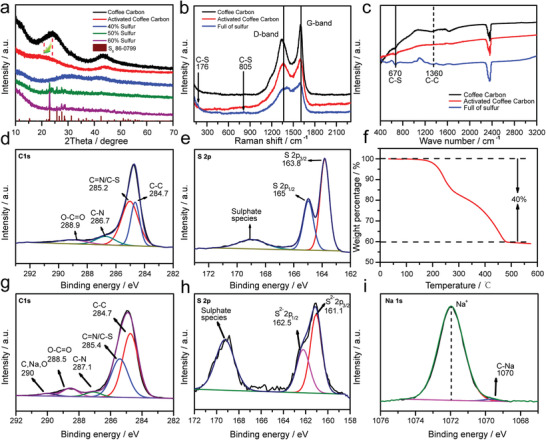
a) XRD patterns of the CC, ACC, ACC‐40S, ACC‐50S, and ACC‐60S samples. b) Raman spectra of the CC, ACC, and ACC‐40S samples. c) FTIR spectra of the CC, ACC, and ACC‐40S samples. d) XPS C 1s core‐level spectrum and e) S 2p core‐level spectrum of the ACC‐40S sample. f) TGA curve of the ACC‐40S sample. g) XPS C 1s core‐level spectrum, h) S 2p core‐level spectrum, and i) Na 1s core‐level of the ACC‐40S composite electrode at the fully discharged state.

X‐ray diffraction (XRD) patterns of CC, ACC, ACC‐40S, ACC‐50S, and ACC‐60S are shown in **Figure 2**a. The characteristic (002) and (004) diffraction peaks of graphite can be observed in the XRD patterns of CC and ACC. It is noticed that the (002) peak of ACC becomes weaker and shifts to lower angle as compared to that of CC, which indicates poorly crystalline feature and expanded interlayer distance due to the increased structure defects from the activation process. ^[^ ^13^ ^]^ Except for the weak diffraction peaks from carbon, the XRD pattern of ACC‐40S does not show any diffraction peaks of S_8_, demonstrating only small sulfur molecules are confined in the micropores without large S_8_ molecules at surface. In contrast, the diffraction peaks of S_8_ phase (JCPDS No. 85‐0799) can be detected in the XRD patterns of the ACC‐50S and ACC‐60S, indicating that only about 40 wt% sulfur can be loaded in the micropores and further increased sulfur content will result in large S_8_ molecules at the surface without micropore confinement. In addition, the S_8_ diffraction peaks still cannot be detected in the ACC‐40S electrode after several charge/discharge cycles, as shown in Figure S5 (Supporting Information), demonstrating the sulfur confinement in the micropores is very stable. The Raman spectra of CC, ACC, and ACC‐40S were further compared in Figure 2b. It is well known that the intensity ratio of D band to G band (*I*_D_/*I*_G_) is an indicator of the defect degree in carbonaceous materials.^[^ ^14^ ^]^ As shown in Figure 2b, the *I*_D_/*I*_G_ ratio of ACC (0.94) is much higher than that (0.86) of CC due to the structure distortion induced by the activation process, which is consistent with the XRD results. It is worth noting that the two peaks located at 176 and 805 cm^−1^ are observed in the Raman spectrum of ACC‐40S, which could be ascribed to the chemical bonds between sulfur and carbon.^[^ ^15^ ^]^ To further investigate the chemical constitutions of CC, ACC, and ACC‐40S samples, Fourier transform infrared spectroscopy (FTIR) measurements were carried out and the obtained FTIR spectra are shown in Figure 2c. In comparison, the FTIR spectrum of ACC‐40S shows extra small peak as compared to those of CC and ACC. The absorption peak at 670 cm^−1^ can be ascribed to C–S group,^[^ ^15^, ^16^ ^]^ which is in good agreement with the Raman results.

X‐ray photoelectron spectroscopy (XPS) measurements were carried out to investigate the chemical species of the CC, ACC, and ACC‐40S samples. As shown in Figure S6a,b (Supporting Information), the XPS survey scan spectra indicate the presence of C, N, and O elements in both CC and ACC samples. Four elements of C, N, O, and S can be detected in the XPS survey scan spectrum of ACC‐40S and Na signal can be further detected when the ACC‐40S electrode is fully discharged (Figure S6c,d, Supporting Information). As shown in Figure 2d, the C 1s core‐level XPS spectrum of ACC‐40S can be deconvoluted into four components at 284.7, 285.2, 286.7, and 288.9 eV, which can be attributed to C─C, C─S/C═N, C─N, and O─C═O groups^[^ ^17^, ^18^ ^]^ respectively, suggesting the N‐containing groups on the carbon surface and the chemical bonding between sulfur and carbon. Such interaction between sulfur and carbon could be beneficial to effectively immobilize the sulfur in the carbon micropores. Figure 2e shows the S 2P core‐level XPS spectrum of the ACC‐40S sample. Two peaks located at 163.8 and 165.0 eV can be assigned to S 2p_3/2_ and 2p_1/2_, respectively. The slightly lower binding energy of S 2p_3/2_ of ACC‐40S than that of pure S powder suggests the interaction between sulfur and carbon,^[^ ^19^ ^]^ which is consistent with Raman and FTIR results. Furthermore, a minor peak at 168.9 eV is ascribed to the sulfate species formed by partial oxidation of sulfur in air.^[^ ^14^ ^]^ The N 1s core‐level XPS spectrum is shown in Figure S7a (Supporting Information), in which three components can be assigned to pyridinic N (399.7 eV), pyrrolic N (400.9 eV), and graphitic N (402.8 eV),^[^ ^18^, ^20^ ^]^ respectively. The high‐resolution XPS spectrum of O 1s in Figure S7b (Supporting Information) exhibits three components at 531.8, 532.6, and 533.8 eV, which can be assigned to C═O, C─O, and ─OH species,^[^ ^21^ ^]^ respectively. After the ACC‐40S electrode is fully discharged, a new peak located at 290 eV emerges in the core‐level XPS spectra of C 1s (Figure 2g), which can be ascribed to the interaction between carbon, sodium, and oxygen.^[^ ^22^ ^]^ In addition, the core‐level XPS spectra of Na 1s (Figure 2i) shows an asymmetric peak, which can be deconvoluted into two components. The major one is assigned to Na^+^ in Na_2_S and the minor one can be attributed to the C─Na bond. This indicates that the sodium ions from Na_2_S have interaction and form a new bond with carbon,^[^ ^23^ ^]^ and such covalent bond can improve the electrical conductivity of Na_2_S. Figure 2h shows the S 2P core‐level XPS spectrum of the fully discharged ACC‐40S electrode. The three peaks at 167.7, 162.5, and 161.1 eV can be assigned to sulfate species, S^2−^ 2P_1/2_, and S^2−^ 2p_3/2_,^[^ ^24^ ^]^ respectively, indicating that Na_2_S is the only discharge product. The binding energy of S^2−^ 2p_3/2_ is slightly higher than 160.6 eV of bulk Na_2_S,^[^ ^24^ ^]^ which could be due to the partial charge‐transfer effect between the S^2−^ anion and carbon in the micropore.^[^ ^25^ ^]^ A strong peak of the sulfate species is also found in the core‐level XPS spectra of S 2p. Although most of sulfur infused into the micropores are stable during the charge and discharge, it is still possible that a small amount of sulfur could come out to the surface, which can be easily oxidized and generate the sulfate species at the surface. Since XPS is especially effective to investigate the surface chemistry, such small amount of sulfate species on the surface could induce obvious signals. As shown in Figure S7g,h (Supporting Information), the core‐level XPS spectra of N 1s shows similar peaks comparing to the ACC‐40S but a new Na Auger peak appears at the discharge state in the high‐resolution XPS spectrum of O 1s.^[^ ^22^ ^]^ For comparison, the core‐level XPS spectra of O 1s, N 1s, and C 1s of CC and ACC are shown in Figures S7 and S8 (Supporting Information), which are similar to those of ACC‐40S. To investigate the interaction between Na_2_S and each functional group, we add one type of functional group in monolayer graphene model every time separately, as shown in Figure S9 (Supporting Information). Here, we added the optimized models of functionalized graphene, and calculated the adsorption energies of Na_2_S on pristine and functionalized graphene (Table S2, Supporting Information). It is found that the functional groups on graphene have strong interaction with Na_2_S molecule by forming O─S, N─S bonds. Especially, if the ─OH can stay inside of the pores and Na_2_S molecule is put nearby, the ─OH will detach from the carbon surface and form a new product of Na_2_SOH (as shown in Figure S9c in the Supporting Information). However, such O─S, N─S bonds, and Na_2_SOH (S^−1^) cannot be detected in our XPS results, indicating such functional groups are negligible inside the pores. On the other hand, the existence of C─O and C─OH bonds can induce the lattice distortion of carbon and cause the surface roughness from 0.6 to 1.1 nm,^[^ ^26^ ^]^ which is not feasible inside the micropores with major pore size of 0.5 nm for the present work. To rule out the effect of coffee origin on the experimental results, we collected some coffee residue from USA and prepared the porous carbon by using the same synthesis procedure. As shown in Figure S10 (Supporting Information), the obtained carbon sample from USA coffee residue exhibits the same microporous structure from BET results and similar N, C, O compositions from XPS results. The thermogravimetric analysis (TGA) of ACC‐40S in Figure 2f reveals that the ACC‐40S sample has 40 wt% sulfur in the composite.^[^ ^18^ ^]^

**Figure 3:**
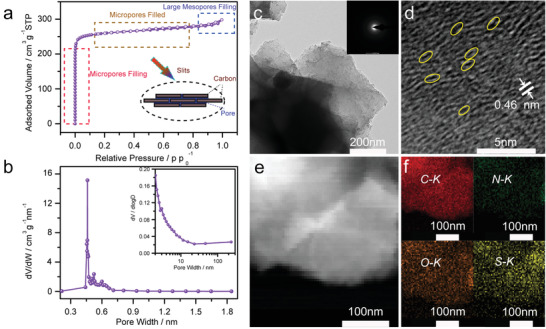
a,b) Nitrogen absorption/desorption isotherms and relative pore size distribution of ACC. c) TEM and d) HRTEM images of the ACC‐40S sample. Inset in (c) is the corresponding SEAD pattern. e,f) STEM image of the ACC‐40S sample and corresponding EDS mappings of C, N, O, and S elements.

The pore structure of ACC is further characterized by nitrogen adsorption/desorption isotherms. **Figure 3**a shows a typical Type I isotherm with H4 reversible hysteresis loop, indicating the existence of a large amount of micropores. The specific surface area of ACC was calculated to be 1100 m^2^ g^−1^ by using the Brunauer−Emmett−Teller (BET) method. It is noted that the adsorption increases sharply when *p*/*p*_0_ is below 0.02 (due to micropore filling) and then the adsorption curve becomes nearly horizontal, which indicates the ultramicropores are slits as illustrated in Figure 3a.^[^ ^27^ ^]^ A hysteresis loop at *p*/*p*_0_ above 0.95 associates with the presence of large mesopores arising from textural mesoporosity.^[^ ^28^ ^]^ The micropore size distribution and the cumulative pore volume distribution calculated by Horvaih–Kawazoe (HK) method is presented in Figure 3b and Figure S11 (Supporting Information). The pore‐size distribution curve reveals that micropores in ACC mainly distribute at around 0.46 nm. The cumulative pore volume for the ultramicropores < 0.7 nm is calculated to be 0.384 cm^3^ g^−1^ for ACC, which corresponds to a theoretical loading of 44 wt% sulfur in micropores. Except for micropores, widely distributed secondary mesopores ranging from 30 to 200 nm could be observed in the pore size distribution curve (inset of Figure 3b), which is consistent with the nitrogen adsorption–desorption isotherms.^[^ ^29^ ^]^ Such mesopores in ACC can facilitate the access of sulfur to the carbon surface and complete infusion of sulfur into the micropores. The morphology and microstructure of the ACC‐40S were further investigated by transmission electron microscopy (TEM). As shown in Figure 3c, translucent carbon nanosheets can be observed from the low‐magnification TEM image of the ACC‐40S, which indicates the 3D porous ACC channel wall is very thin. The selected area electron diffraction (SAED) pattern in Figure 3c reveals low degree of crystallinity of ACC‐40S because all of the sulfur is confined in the ultramicropores. High‐resolution transmission electron microscopy (HRTEM) image in Figure 3d reveals the slit type ultramicropores, which is in good agreement with the BET results. For the control group, we did HRTEM for an amorphous carbon nanosheet sample (Figure S12, Supporting Information), which has similar surface area with ACC. The HRTEM of the amorphous carbon nanosheet is compared with the HRTEM for our microporous carbon. In comparison, we can see the textures of the two samples are different, and the slit‐shape features cannot be observed in the HRTEM image of the amorphous carbon nanosheet sample. The scanning transmission electron microscopy (STEM) image and corresponding energy‐dispersive X‐ray spectroscopy (EDS) element mappings reveal a uniform distribution of sulfur in the ACC‐40S (Figure 3e,f), which suggests that the short‐chain‐like sulfur molecules including S_2–4_ are well dispersed in the ultramicropores of ACC.

**Figure 4:**
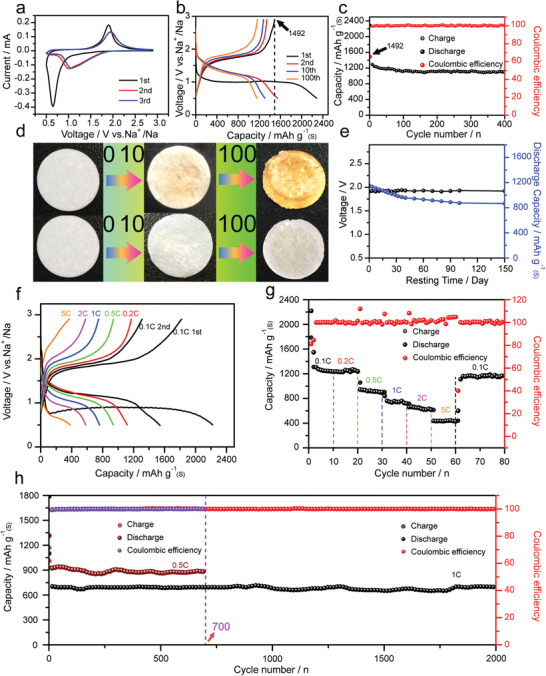
a) CV curves of the ACC‐40S electrode at a scan rate of 0.1 mV s^−1^. b) Discharge/charge curves of the ACC‐40S electrode at 0.1 C. c) Cycle performance of the ACC‐40S electrode at 0.1 C for 400 cycles. d) The photo images of the separators from the cells using CAC‐40S and ACC‐40S as the cathodes after different cycle numbers. e) Open‐circuit voltage and capacity retention of the cell over 150 days resting period. f) Discharge/charge curves of the ACC‐40S electrode at varied current densities. g) Specific capacities of the ACC‐40S electrode at varied current densities. h) Cycle performances of the ACC‐40S electrode at 0.5 and 1 C for 700 and 2000 cycles, respectively.

The electrochemical properties of RT Na–S batteries using ACC‐40S as cathode were evaluated through the CR2032‐type coin cells. The cyclic voltammetry (CV) curves of the ACC‐40S electrode at a scan rate of 0.1 mV s^−1^ between 0.5 and 2.9 V for the initial three cycles are shown in **Figure 4**a. As shown in Figure 4a, the first cathodic process (sodiation) exhibits a strong peak at around 0.7 V, which is lower than 1.1 V for the following cycles. The lower‐voltage cathodic peak for the first cycle was also reported in previous works, suggesting an activation process associated with Na^+^ diffusion into the micropores.^[^ ^3^, ^30^ ^]^ Different from previous works, there is only one cathodic peak for all cycles, corresponding to the direct formation of Na_2_S. For the anodic scan, the peak at around 1.9 V can be assigned to the reverse oxidation process to extract Na^+^ from the electrode.^[^ ^31^ ^]^ The CV curves of the ACC‐40S electrode at different scan rates of 0.1, 0.3, 0.5, and 1 mV s^−1^ are revealed in Figure S13 (Supporting Information). The reduction peaks are seen to shift toward lower voltage while the oxidation peaks move toward higher voltage with increasing scan rate, which can be to the increased polarization for the cell at high scan rates.

Figure 4b shows discharge/charge curves of the ACC‐40S electrode at the 1st, 2nd, 10th, and 100th cycles between 0.5 and 2.9 V at 0.1 C. The discharge curve shows a sloppy voltage plateau between 1.0 and 1.7 V and the charge curve shows a voltage plateau at around 1.7 V, indicating single‐step redox reaction as revealed in CV results. As shown in Figure 4c, the ACC‐40S electrode delivers a high reversible specific capacity of 1492 mAh g^−1^ for the first cycle and can still remain 1110 mAh g^−1^ after 400 cycles. Even when the electrode loading of the ACC‐40S electrode was increased up to 3 mg cm^−2^, it can still deliver a high reversible capacity of 960 mAh g^−1^ at 0.1 C after 200 cycles (Figure S14, Supporting Information). The discharge/charge curves and cycle performances of the ACC‐*x*S (*x* = 50, 60, 70, and 80) electrodes with high sulfur loadings are shown in Figure S15 (Supporting Information) for comparison. As the micropores in the current carbon material can only accommodate about 44 wt% sulfur, extra sulfur loading can proceed through the carbon surface adsorption. However, the surface adsorbed sulfur is easy to get lost due to polysulfide formation and dissolution during the charge/discharge processes.^[^ ^32^ ^]^ Accordingly, the ACC‐50S and ACC‐60S electrodes show much lower specific capacities of 816 and 705 mAh g^−1^, respectively, after 400 cycles. However, with a high sulfur loading of ≈70 wt%, the ACC‐70S electrode shows a visible plateau at around 1.9 V during the first discharge process, indicating the generation of polysulfides. When the sulfur loading reaches 80 wt%, such high voltage plateau is more pronounced in the first discharge curve, suggesting severe formation of polysulfides. Obviously, the cell performance is significantly deteriorated by the high sulfur loading and formation of massive polysulfide. Both ACC‐70S and ACC‐80S electrodes exhibit very low specific, indicating very low utilization of sulfur in the electrodes. For further comparison, the CC‐40S and commercial activated carbon–sulfur (CAC‐40S) electrode were also tested under the same conditions (Figure S16, Supporting Information). The charge/discharge curves of both the CC‐40S and CAC‐40S electrodes show very low reversible capacities, indicating very low utilization of sulfur in both electrodes.^[^ ^33^ ^]^

Since the separator is in close contact with the cathode, the color change of the separator during cycling can be used as an indicator, reflecting the formation and dissolution of sodium polysulfides from the cathode.^[^ ^34^ ^]^ The photo images of the separators in the cells using CAC‐40S (the first row) and ACC‐40S (the second row) as cathodes after 0, 10, and 100 cycles are shown in Figure 4d. It is obvious that the color of separators in the first row turns from white to dark yellow after 100 cycles. In contrast, the separators in the second row retain the white color, suggesting negligible polysulfides generation in the ACC‐40S electrode during cycling. With polysulfides formation and the shuttling effect, the fast self‐discharge could be a serious problem for both Li–S and Na–S batteries. In the present study, the charge/discharge behavior and the separator color change have demonstrated that polysulfides formation can be greatly suppressed in the ACC‐40S electrode. Therefore, the self‐discharge of the ACC‐40S electrode was further investigated for the RT Na–S batteries. As shown in Figure 4e, the black line displays the time‐dependent open‐circuit voltage (OCV) profile of the Na–S battery over the rest time of 150 days. It is surprising to see that the OCV only drops from 1.92 to 1.91 V over the 150 days’ resting period, suggesting extremely low self‐discharge for the present Na–S battery. The blue line in Figure 4e displays the time‐dependent capacity‐retention over the same rest period. Overall, there is about 25.5% capacity loss over 150 days’ rest, corresponding to a capacity fading rate of 0.17% per day. As shown in Figure S17 (Supporting Information), the present ACC‐40S electrode presents much lower self‐discharge rate as compared to other electrodes for both Li–S and Na–S batteries reported in literature. Such a low self‐discharge rate of the ACC‐40S electrode indicates that micropore confinement is effective to prevent polysulfide formation and this Na–S system possesses outstanding static electrochemical stability.

To further investigate the rate performance, the charge/discharge measurements of the ACC‐40S electrode were carried out at different current densities from 0.1 to 5 C. As shown in Figure 4f,g, the average specific capacities of the ACC‐40S electrode are 1240, 1233, 900, 720, 620, and 423 mAh g^−1^ at 0.1, 0.2, 0.5, 1, 2, and 5 C respectively, demonstrating good rate capability. Notably, when the current density returns to 0.1 C, a reversible capacity of 1170 mAh g^−1^ could still be recovered, indicating high reversibility for the electrode. The superior rate performance of the ACC‐40S electrode can be attributed to its unique 3D structure, facilitating electrolyte penetration, and small sulfur molecules confined in the micropores, favoring fast reaction kinetics. It is worth noting that the ACC‐40S electrodes exhibits superb cycling stability even at high current densities. As shown in Figure 4h, the ACC‐40S electrode can achieve 90% capacity retention after 700 cycles at 0.5 C and 95% capacity retention after 2000 cycles at 1 C. The ultrastable cycling stability and long cycle life of the ACC‐40S electrode indicate that micropore confinement could be an effective strategy for developing highly stable cathode materials for RT Na–S batteries.

**Figure 5:**
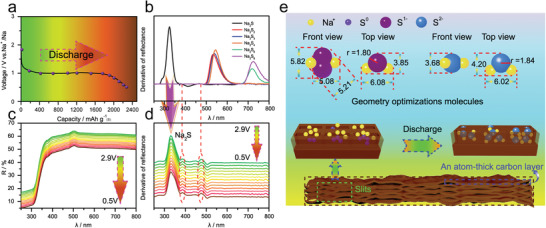
a) Discharge profile of the ACC‐40S electrode at 0.1 C. b) UV/vis spectra first‐order derivative curves of different Na_2_S*_x_* (0 ≤ *x* ≤ 8) products as reference. c) In situ UV/vis spectra and d) homologous first‐order derivative curves of the ACC‐40S electrode. e) Schematic illustration of the reaction mechanism of the ACC‐40S cathode for RT Na–S batteries.

In order to investigate the reaction mechanism and the reaction products during battery cycling, in situ UV/vis spectroscopy measurements were performed at different discharge states as shown in **Figure 5**a.^[^ ^35^ ^]^ As reference, the UV/vis spectra first‐order derivative curves of different Na_2_S*_x_* (2 ≤ *x* ≤ 8) products are displayed in Figure 5b. Six different products were prepared with different Na/S ratios and dissolved in triethylene glycol dimethyl ether (TEGDEM), and the color of the solvent changes from transparency to dark red with increasing sulfur content as shown in Figure S18 (Supporting Information). Figure 5c,d shows the in situ UV/vis spectra and corresponding first‐order derivative curves for the cell at different discharge states. It is worth noting that only one derivative peak from Na_2_S can be detected without any peak shifting during the whole discharge process, demonstrating that Na_2_S is the only product during discharge.^[^ ^36^ ^]^ The in situ UV/vis spectroscopy can detect the dissolved Na_2_S and polysulfides in the electrolyte. The electrolyte we used in this study is 1.0 m NaClO_4_ in ethylene carbonate (EC)/propylene carbonate (PC), and the solubility of Na_2_S in this electrolyte is very low as compared to those of the dissoluble polysulfides. Due to the very low solubility of Na_2_S, the UV signal of Na_2_S dissolved in the electrolyte does not change much, and cannot reflect the variation of Na_2_S in the electrode. Neither soluble polysulfides nor insoluble polysulfides can be formed during the discharge process in the ACC‐40S electrode, which agrees well with electrochemical behavior, indicating one‐step redox reaction for the present Na–S system. In order to ensure the reversibility of the cell, in situ UV/vis spectra for the cell were also measured at different voltages during the charge process (Figure S19, Supporting Information), which indicates sulfur can be the only charge product without polysulfides formation. Therefore, it can be confirmed that no polysulfides (Na_2_S*_x_*, 2 ≤ *x* ≤ 8) can be formed during the whole reaction process for ACC‐40S electrode, and this new one‐step reaction mechanism could be the main reason resulting in the ultrastable cycling for the RT Na–S batteries.

**Figure 6:**
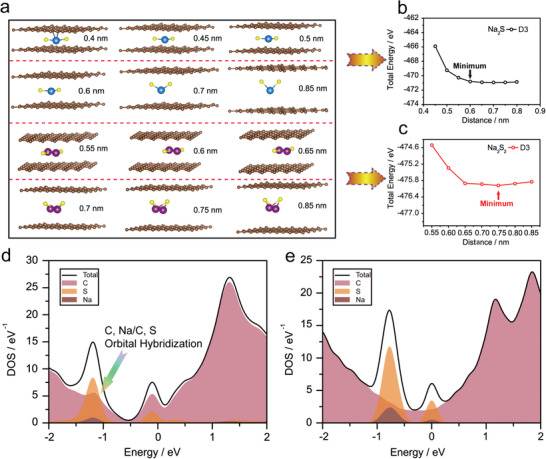
a) Optimized molecule structures of Na_2_S and Na_2_S_2_ confined into the bilayer graphene with different interlayer spacings. Total energy profiles of b) Na_2_S and c) Na_2_S_2_ confined into the bilayer graphene with interlayer distance changing from 0.45 to 0.85 nm. The calculated partial density of states (PDOS) of Na_2_S confined in bilayer‐graphene with d) 0.5 nm and e) 0.8 nm interlayer spacings.

To further understand the one‐step reaction of the present Na–S battery without polysulfides formation, the reaction mechanism of the ACC‐40S electrode with geometry consideration for micropores is illustrated in Figure 5e. It can be seen that the free Na_2_S_2_ molecule has a 3D structure, while the Na_2_S molecule is 2D, and this difference is the key factor that makes Na_2_S easier to form in the slit micropores. The possible reaction mechanism for converting small sulfur molecules S*_n_* (*n* = 2–4) into Na_2_S is proposed and shown in Figure S20 (Supporting Information). **Figure** **6**a shows the optimized structures of Na_2_S and Na_2_S_2_ molecules intercalated into the bilayer graphene (simulating the slit micropore) with different interlayer spacings. Na_2_S with a plane structure can be laying in the narrow slit even when the interlayer spacing is less than 0.5 nm without changing its molecular configuration. Since the interaction between Na_2_S and the carbon‐layer (up and down) is van der Waals (vdW) force, from Figure 6b we find the total energy has a minimum at an interlayer spacing of 0.6 nm, and the distance from Na_2_S to the up/down carbon layer is about 0.3 nm, which is a typical distance for vdW interactions. With the decreasing of interlayer spacing, the repulsion between Na_2_S and carbon layers increases, which leads to the enhancement of total energy. When the interlayer spacing is larger than 0.60 nm, the total energy of the system also slightly increases, because the increasing of distance between Na_2_S and up carbon layer weakens the interaction, which indicates Na_2_S molecules are free to form in the interlayer when interlayer distance is larger than 0.60 nm.

In case of Na_2_S_2_, a larger interlayer distance is required to keep its corrugated configuration. Seen in Figure 6a, it is noted that the 3D Na_2_S_2_ molecule will be distorted when the interlayer spacing is less than 0.75 nm. As shown in Figure 6c, the total energy has minimum at 0.75 nm, thus Na_2_S_2_ could form when the interlayer distance is larger than 0.75 nm. Considering the micropore size distribution is dominated at about 0.46 nm and the pore size distribution above 0.75 nm is nearly zero in our ACC, the Na_2_S_2_ molecules are hardly to form in the slit micropores from the thermodynamic analysis, agreeing well with our experimental results. Therefore, as a result of micropore confinement, Na_2_S is the only product for the Na–S system, enabling the one‐step reaction mechanism without polysulfides formation. The optimized structures of Na_2_S and Na_2_S_2_ molecules intercalated into the bilayer graphene with other interlayer spacings are also shown in Figure S21 (Supporting Information). Although Xin et al. thought that both Na_2_S_2_ and Na_2_S molecules can be accommodated as single molecule in the cylindrical micropores with 0.5 nm diameter, the difference may be induced by the effect of curvature, because they used a cylindrical micropore model.

When the interlayer spacing is less than 0.45 nm, which is close to bonding distance, the strong repulsion causes sharply increased total energy of the system. When the interlayer distance ranges from 0.45 to 0.60 nm, Na_2_S molecules still can exist, but the charge transfer from Na_2_S to carbon layer has been enhanced and the probability to form Na─C/S─C bonds are raised. The partial the density of states (PDOS) of the systems with one Na_2_S molecule in narrow slit with 0.5 and 0.8 nm distance are shown in Figure 6d,e, respectively. As shown in Figure 6e, only orbital hybridization between sulfur and sodium can be found, which means that Na_2_S maintains a molecular structure. The interaction between Na_2_S and slits is van der Waals forces. While for Figure 6d, orbital hybridization between Na and C (C and S) appears, which indicates the formation of Na─C (C─S) bonds. Through analysis of the results of XPS we do find out the existence of C─Na bond, the C─Na could promote the oxidation of Na_2_S during discharging. The strong interaction between Na_2_S and carbon layer improves the charge transfer from Na_2_S to carbon layer and enhances the carrier concentration of carbon layer. As the electronic conductivity is proportional to carrier concentration, the confinement of Na_2_S molecules into the slit micropores is beneficial to improve the electron transfer, thus further increasing the reaction efficiency.

## 3 Conclusion

In summary, we report the preparation of coffee residue derived 3D microporous carbon with small sulfur molecules (S_2–4_) confined in the slit micropores by tuning the infused sulfur content. The electrochemical behavior of the carbon–sulfur composite electrode with 40% sulfur content (ACC‐40S) indicates one‐step Na–S reaction, and the in situ UV/vis spectroscopy result demonstrates Na_2_S is the only discharge product without formation of polysulfides during charge/discharge for the Na–S batteries. As a result, the ACC‐40S electrode exhibits a high reversible specific capacity up to 1492 mAh g^−1^ at 0.1 C, good rate capability, low‐self discharge, and excellent cycling stability with negligible capacity loss at 1 C after 2000 charge/discharge cycles. The micropore confinement has been elucidated with the DFT calculation results using slit micropore model, demonstrating formation of solo Na_2_S product in the composite electrode is thermodynamically feasible by tailoring proper micropore size. The present work provides new insights for micropore confinement and porous carbon materials design for developing ultrastable RT Na–S batteries without polysulfide formation.

## 4 Experimental Section

##### ACC Preparation

At first, the coffee residue was dried at 100 °C for at least 12 h to ensure there is no water in it. Then 2.5 g dried coffee residue and 1.7 g KOH were mixed in 150 mL solution composed of 100 mL deionized water and 50 mL ethanol, and stirred for 12 h. After that, the collected powder was dried at 100 °C again. The obtained powder was preheated at 200 °C for 1.5 h and then heated at 800 °C for 3 h under Ar atmosphere with a heating rate of 3 °C min^−1^. At last, the sintered product was regulated to neutrality by a 3 m HCl solution and dried at 100 °C for 6 h to obtain the final product.

##### ACC‐*x*S Preparation

The different ACC‐*x*S materials were obtained by a traditional melt diffusion method. Briefly, the ACC was mixed with sulfur in the ratio of 6:4.5, 5:5.5, 4:6.5, 3:7.5, and 2:8.5 (ACC‐*x*S, *x* = 40, 50, 60, 70, and 80) in a mortar for at least 20 min. Then the mixtures were transferred into Teflon containers and preserved at 155 °C for 15 h. At last the black powder was heated at 200 °C for 20 min in Ar atmosphere to obtain the ACC‐*x*S. For comparison, the CC‐40S and CAC‐40S were also prepared through the same preparation process.

##### Structure and Morphology Characterization

The morphologies and microstructures of the samples were investigated by FESEM (Quant 250 FEG) and HRTEM (Tecnai G2 F30 S‐TWIN). Nitrogen adsorption and desorption isotherms were measured with an adsorption apparatus (JW BK‐200C). The crystal structure and phase purity of the as‐prepared samples were characterized using XRD (Bruker‐AXS D8 Advance) and Raman (Jobin‐Yvon T6400 Micro‐Raman system with laser excitation length of 514.5 nm). The sulfur content of the composite was measured by TGA (SDTA851E). The chemical composition and electronic structure of the samples were investigated by XPS (ESCALAB250Xi with a monochromatic Al anode X‐ray source) and FTIR with a Nicolet FTIR Is 10 spectrometer.

##### Electrochemical Measurements

To prepare the electrodes, active materials, super‐P, and poly acrylic acid (PAA) binder were mixed in *N*‐methyl pyrrolidinone with a mass ratio of 8:1:1 to form a homogeneous slurry, and then the slurry was coated on an aluminum foil. The electrodes were dried at 70 °C for 24 h in a vacuum oven. The loading density of the sulfur was about 1 mg cm^−2^. To test the electrochemical performance, the cells were assembled in an argon‐filled glove box in which the water and oxygen concentrations are kept below 0.1 ppm. Here the ACC‐*x*S, CC‐40S, and CAC‐40S were used as working electrodes, Na foil as counter and reference electrode, glass fiber as separator (Whatman GF/F), and 1.0 m NaClO_4_ in EC/(PC) (1:1 by volume) with 2 wt% fluorinated ethylene carbonate (FEC) as electrolyte. The CV measurements were carried out using a CHI660D electrochemical workstation. Charge/discharge measurements were performed in a potential range of 0.5 and 2.9 V (vs Na^+^/Na) at different current densities using LAND CT2001A battery tester.

##### Synthesis of Na_2_S*_x_* Solution and Adsorption Test

Six different sodium polysulfide solutions with varied ratios of sodium to sulfur were prepared, which are denoted according to the sulfur content in Na_2_S*_x_* (*x* = 1, 2, 3, 4, 6, and 8). The sodium sulfide and sulfur with molar ratios of 1:0, 1:1, 1:2, and 1:3 were dissolved in deionized water via magnetically stirring for 2 h to synthesize Na_2_S, Na_2_S_2_, Na_2_S_3_, and Na_2_S_4_, respectively. And the Na_2_S_6_ and Na_2_S_8_ were synthesized with the sodium sulfide to sulfur molar ratios of 1:5 and 1:7 dissolved in TEGDME. The adsorption ability of Na_2_S*_x_* were qualitatively determined using the UV–vis spectrometer and the effect of the solvent would be removed during the testing process.

##### In Situ UV–Vis Spectrometer Measurements

The in situ UV–vis spectroscopy was operated on UV Lambda 750S UV/Vis/NIR spectrometer. The positive case and the sodium metal were punched and the holes were half size of the cathode. 1.0 m NaClO_4_ in EC/PC (1:1 by volume) with 2 wt% FEC was used to infiltrate the separator. As the cathode material, the sulfur loading of the ACC‐40S was about 1 mg cm^−2^. At last, the cell was assembled in a traditional way.

##### Calculation Methods

The geometry optimizations are performed by the Vienna Ab initio Simulation Package (VASP), ^[^ ^37^, ^38^ ^]^ based on DFT. The generalized gradient approximation (GGA) with Perdew–Burke–Ernzerhof (PBE) algorithm is selected as exchange‐correlation interaction.^[^ ^39^ ^]^ To improve the description of long‐range van der Waals interactions, the DFT‐D3 method was employed.^[^ ^40^, ^41^ ^]^ An energy cutoff of 500 eV and a Monkhorst–Pack scheme with a *k*‐point mesh of 11 × 11 × 1 were employed to ensure the calculation accuracy. The structures of bilayer graphene were fixed and Na_2_S and Na_2_S_2_ molecules were relaxed with energy change converged to 1 × 10^−5 ^eV in electronic self‐consistency cycles and all force smaller than 0.02 eV Å^−1^ in ionic relation loops. The PDOS were obtained by the Atomistix ToolKit (ATK) package. ^[^ ^42^ ^]^

## Conflict of Interest

The authors declare no conflict of interest.

## Supporting information

Supporting Information
