# Supplementary material for: Ultrastable Sodium–Sulfur Batteries without Polysulfides Formation Using Slit Ultramicropore Carbon Carrier
Source: Adv Sci (Weinh). 2020 Apr 22;7(11):1903246. doi: 10.1002/advs.201903246 (PMC7284216; doi:10.1002/advs.201903246)
Supplement: Supplementary file 1 — Supporting Information [file ADVS-7-1903246-s001.pdf]

((Supporting Information can be included here using this template))

Copyright WILEY-VCH Verlag GmbH & Co. KGaA, 69469 Weinheim, Germany, 2020.

Supporting Information

Ultrastable Sodium-Sulfur Batteries without Polysulfides Formation Using Slit Ultramicropore Carbon Carrier

Qiubo Guo, Shuang Li, Xuejun Liu, Haochen Lu, Xiaoqing Chang, Hongshen Zhang, Xiaohui Zhu, Qiuying Xia, Chenglin Yan * *and Hui Xia* *

Q. B. Guo, Prof. S. Li, H. C. Lu, X. Q. Chang, H. S. Zhang, X. H. Zhu, Q. Y. Xia, Prof. H. Xia
School of Materials Science and Engineering, Nanjing University of Science and Technology, Nanjing 210094, China.
Q. B. Guo, Prof. S. Li, H. C. Lu, X. Q. Chang, H. S. Zhang, X. H. Zhu, Q. Y. Xia, Prof. H. Xia
Herbert Gleiter Institute of Nanoscience, Nanjing University of Science and Technology, Nanjing 210094, China.
X. J. Liu, Prof. C. L. Yan,
Soochow Institute for Energy and Materials Innovations, College of Energy, Key Laboratory of Advanced Carbon Materials and Wearable Energy Technologies of Jiangsu Province, Soochow University, Suzhou 215006, China.
E-mail: E-mail: c.yan@suda.edu.cn; xiahui@njust.edu.cn

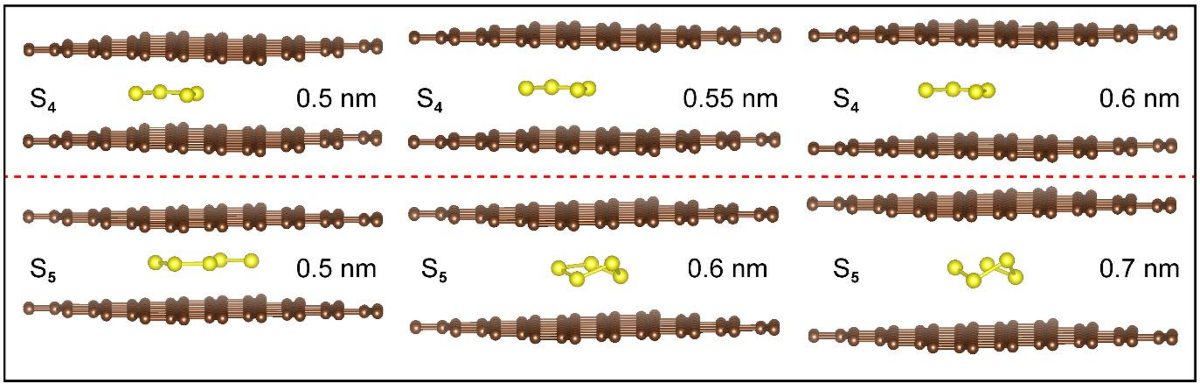

Figure S1. Optimized molecule structures of S_4 and S_5 confined into the bilayer graphene with different interlayer spacings.

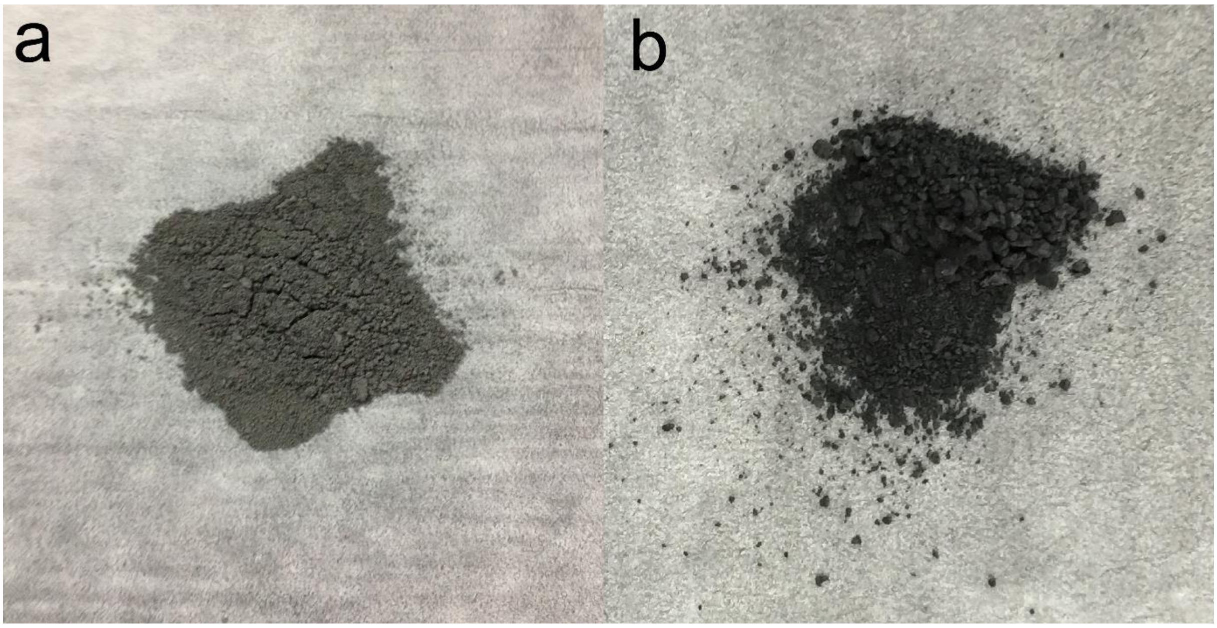

Figure S2. The photo images of ACC-40S before (a) and after (b) heating at 155 °C.

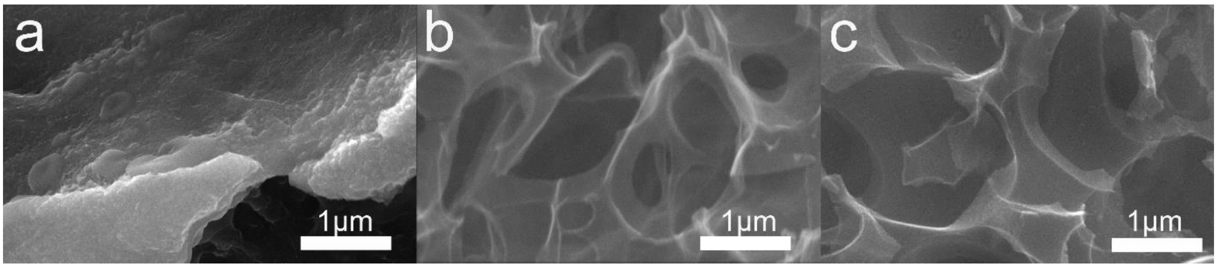

Figure S3. (a-c) FESEM images of the CC, ACC and ACC-40S.

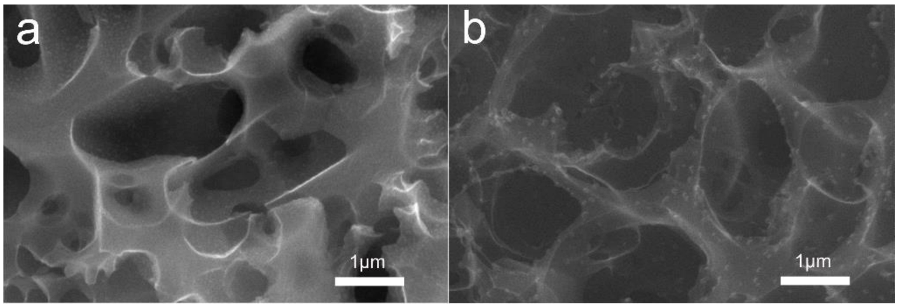

Figure S4. FESEM images of the (a) ACC-50S and (b) ACC-60S.

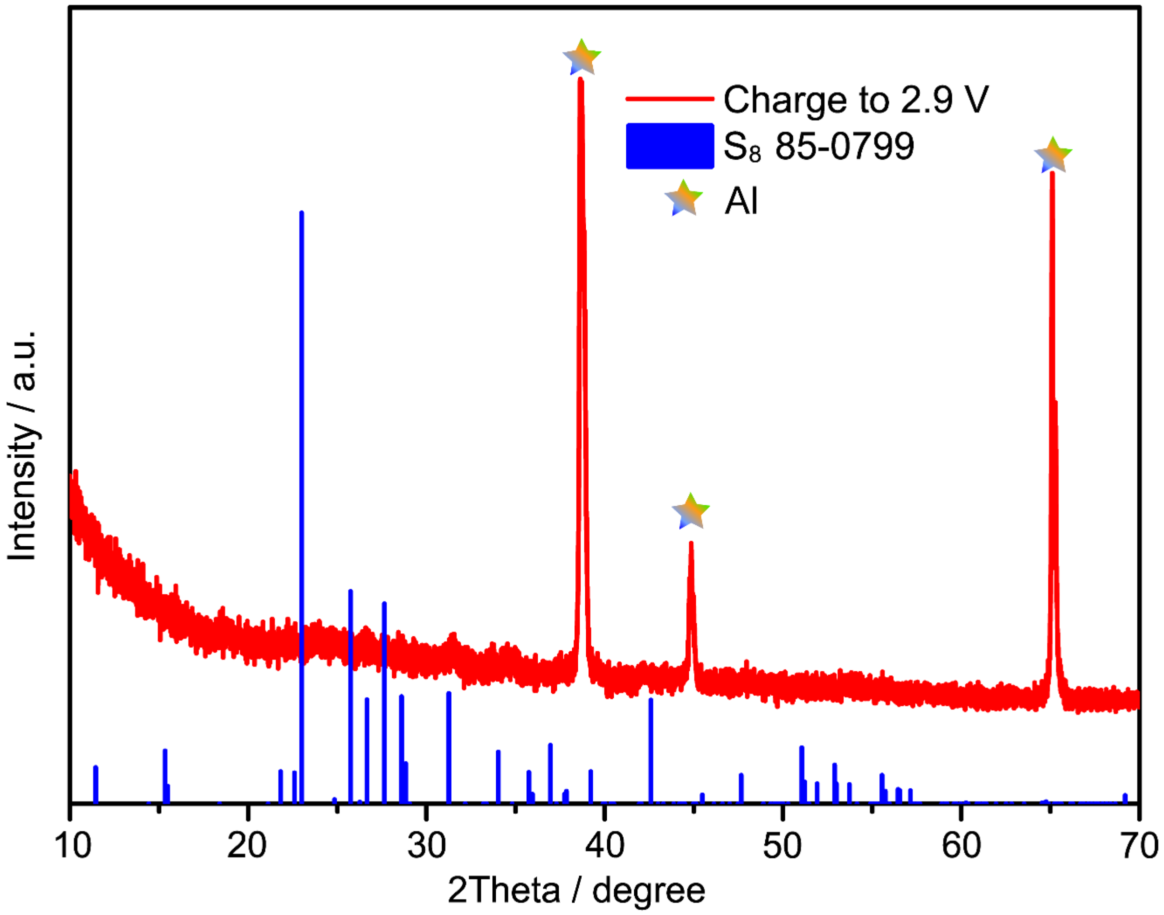

Figure S5. XRD pattern of the ACC-40S sample at the fully charged state after several charge/discharge cycles.

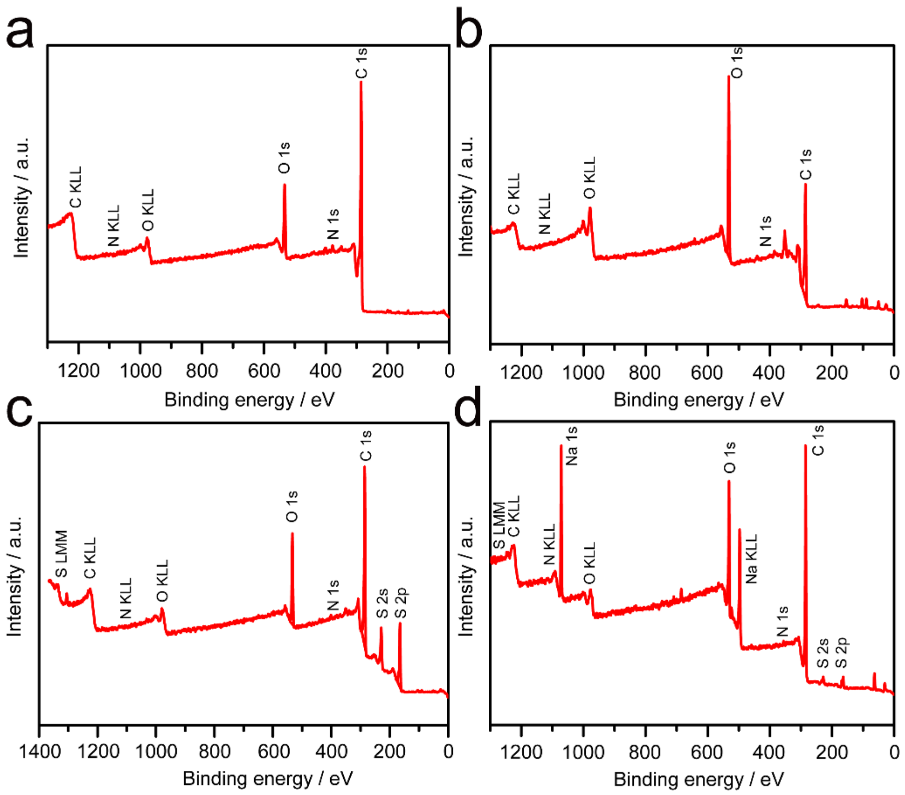

Figure S6. XPS survey scan spectra of the CC, ACC, ACC-40S samples and the ACC-40S at the fully discharged state.

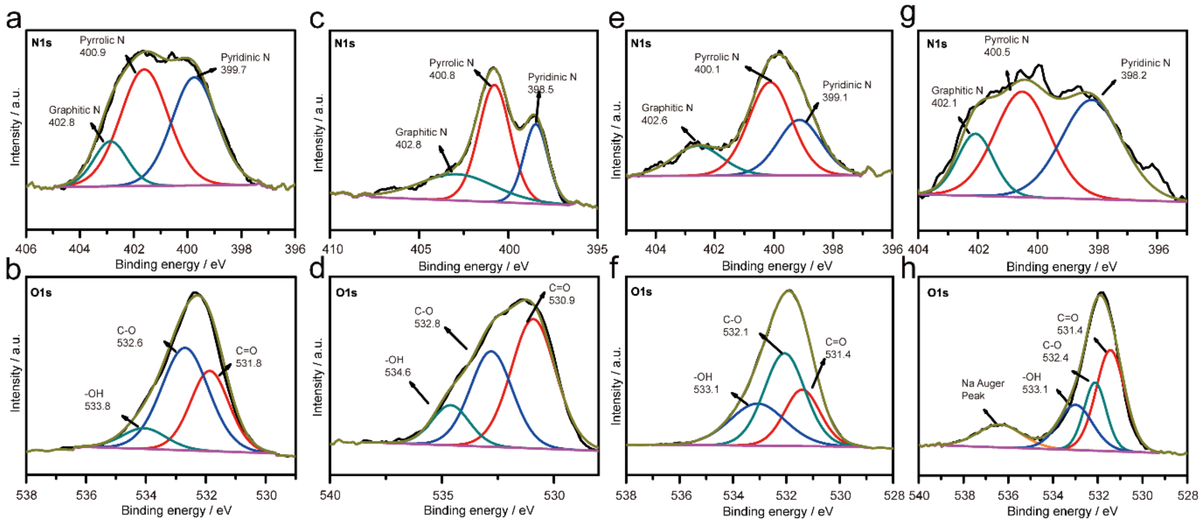

Figure S7. (a, c, e, g) XPS N 1s core-level spectrums and (b, d, f, h) O 1s core-level spectrums of the ACC-40S, CC, ACC and the ACC-40S at fully discharged state.

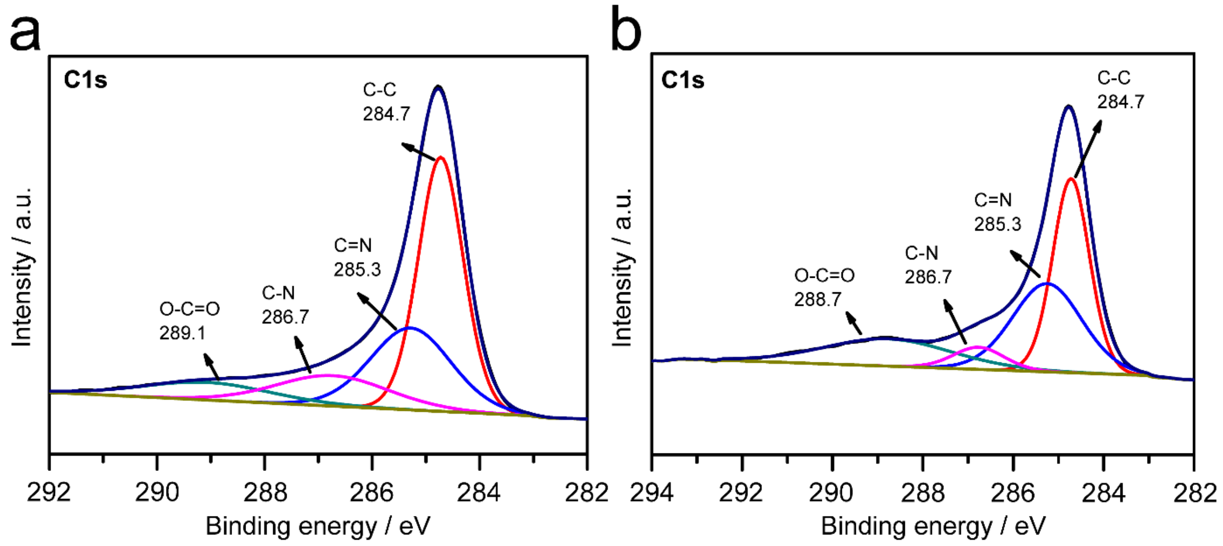

Figure S8. XPS C 1s core-level spectrums of the CC (a) and ACC (b).

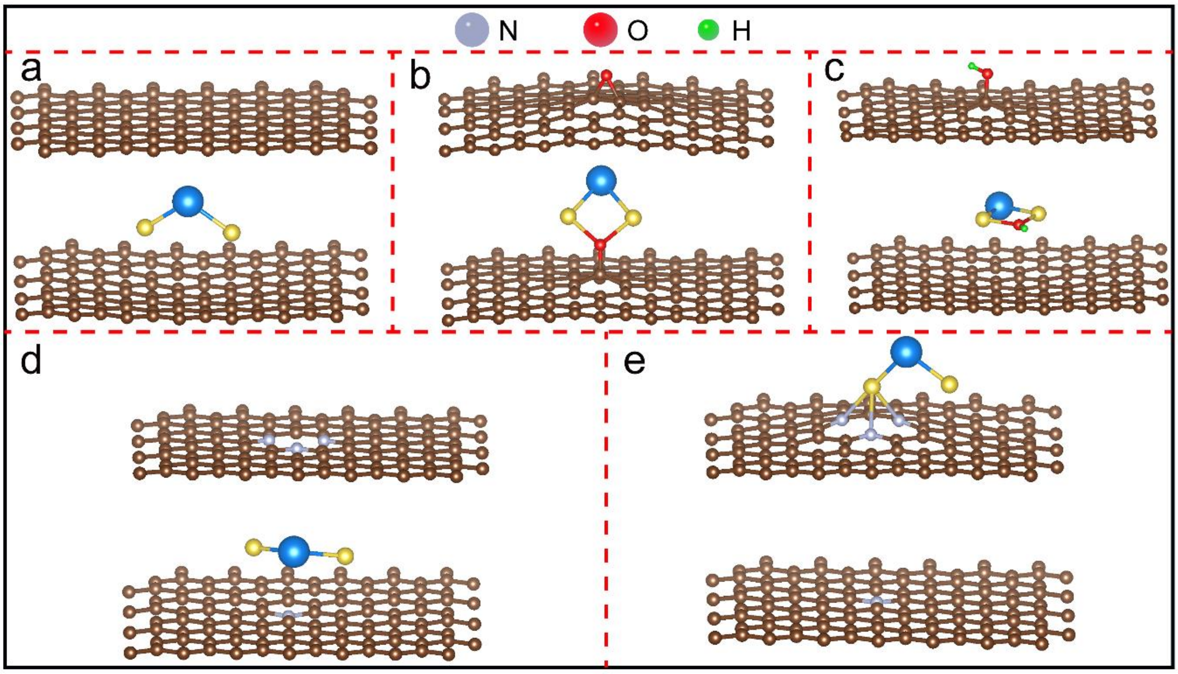

Figure S9. Most stable adsorption configuration of Na_2S on pristine graphene (p-G) and oxygen (O-G), hydroxyl (OH-G), nitrogen (N-G and 3N-G) functionalized graphene obtained from DFT calculations.

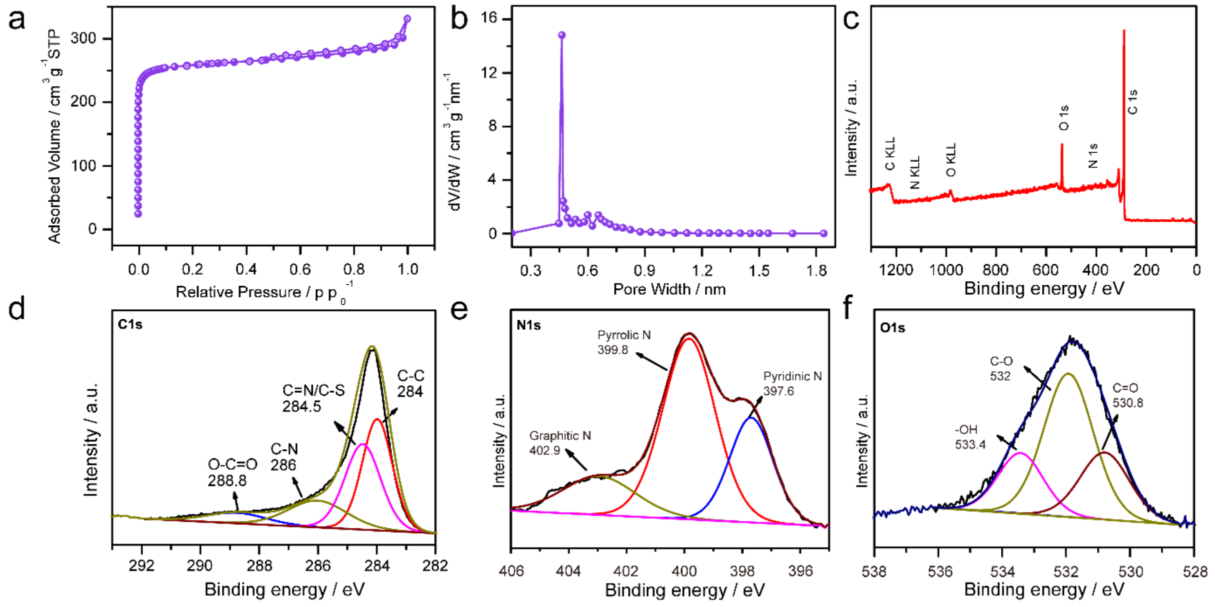

Figure S10. (a, b) Nitrogen absorption/desorption isotherms and relative pore size distribution of ACC obtained from USA coffee residue. (c) XPS survey scan spectra of the ACC obtained from USA coffee residue. (d) XPS C 1s core-level spectrum, (e) N 1s core-level spectrum, and (f) O 1s core-level spectrum of the ACC obtained from USA coffee residue.

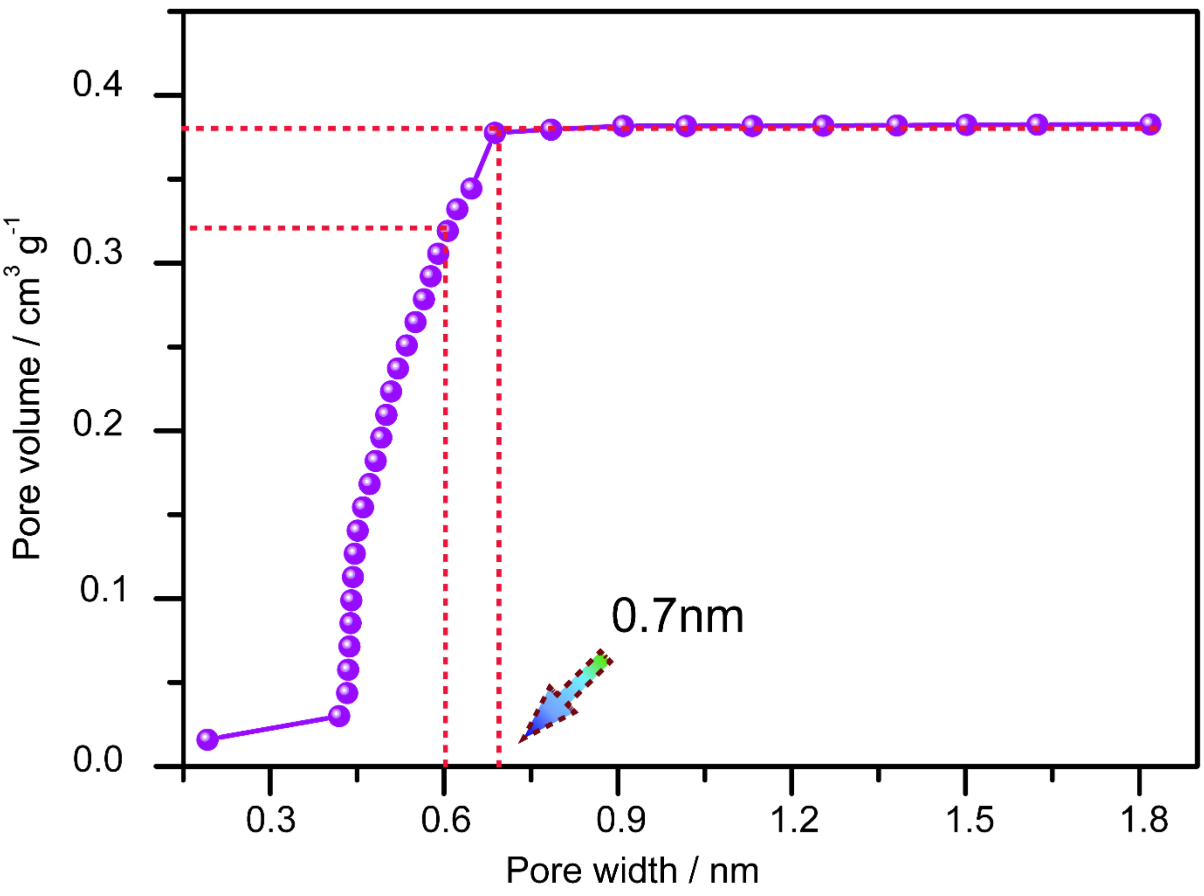

Figure S11. Cumulative pore volume calculated by Horvaih-Kawazoe (HK) model of ACC.

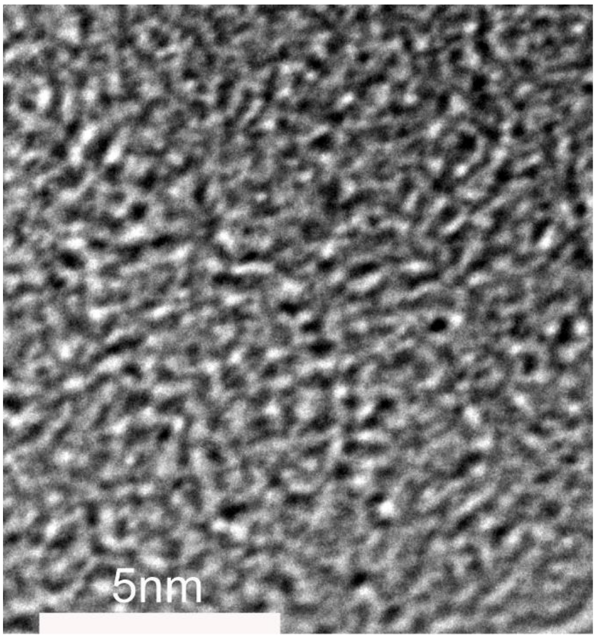

Figure S12. HRTEM images of an amorphous carbon nanosheet sample, which has the similar surface area with ACC sample.

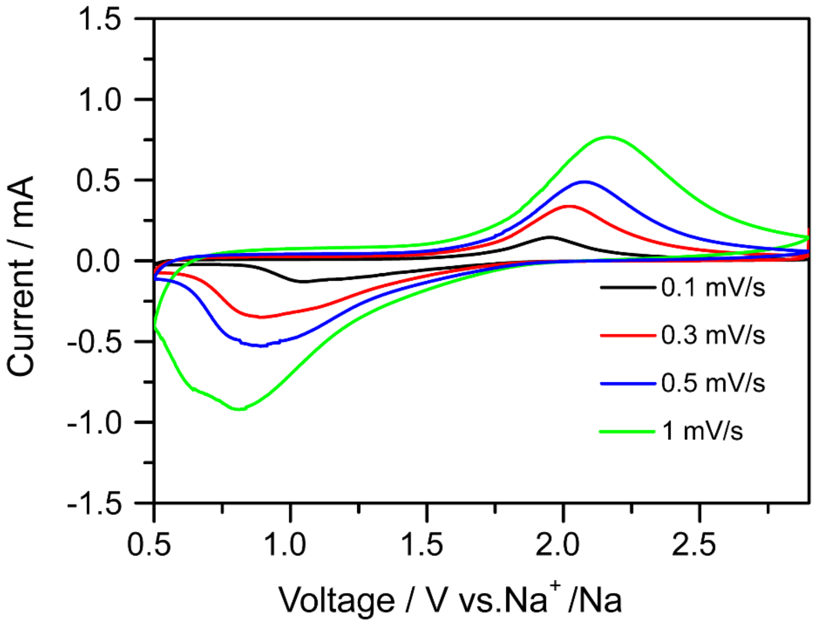

Figure S13. CV curves of the ACC-40S electrode at different scan rates.

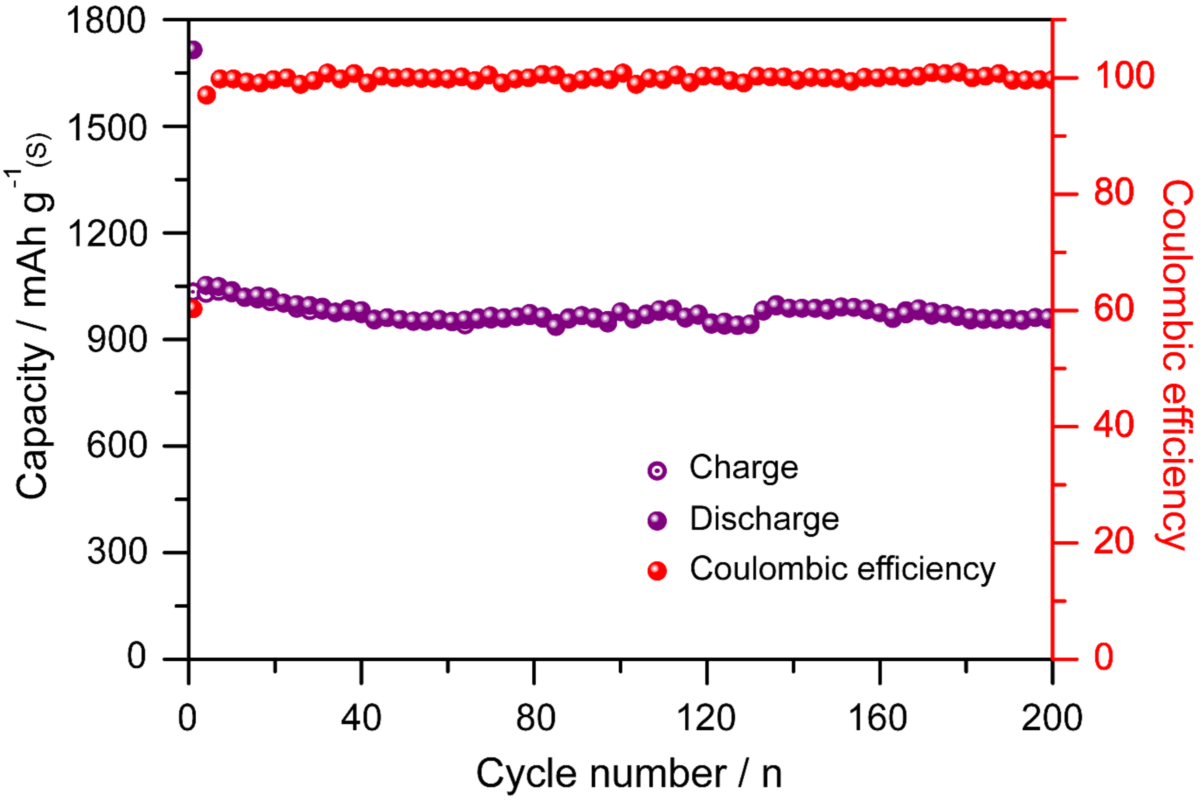

Figure S14. Cyclic performance of the ACC-40S electrode at 0.1C with a mass loading of 3 mg cm^{-2}.

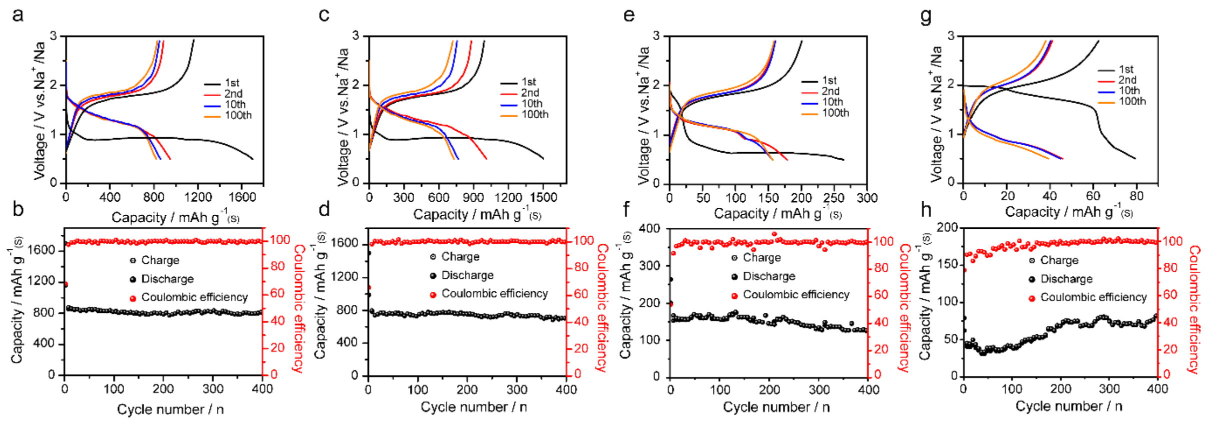

Figure S15. (a, c, e, g) Discharge/charge curves of the ACC-50S, ACC-60S, ACC-70S and ACC-80S electrodes at 0.1 C. (b, d, f, h) Cycle performances of the ACC-50S, ACC-60S, ACC-70S, and ACC-80S electrodes at 0.1 C.

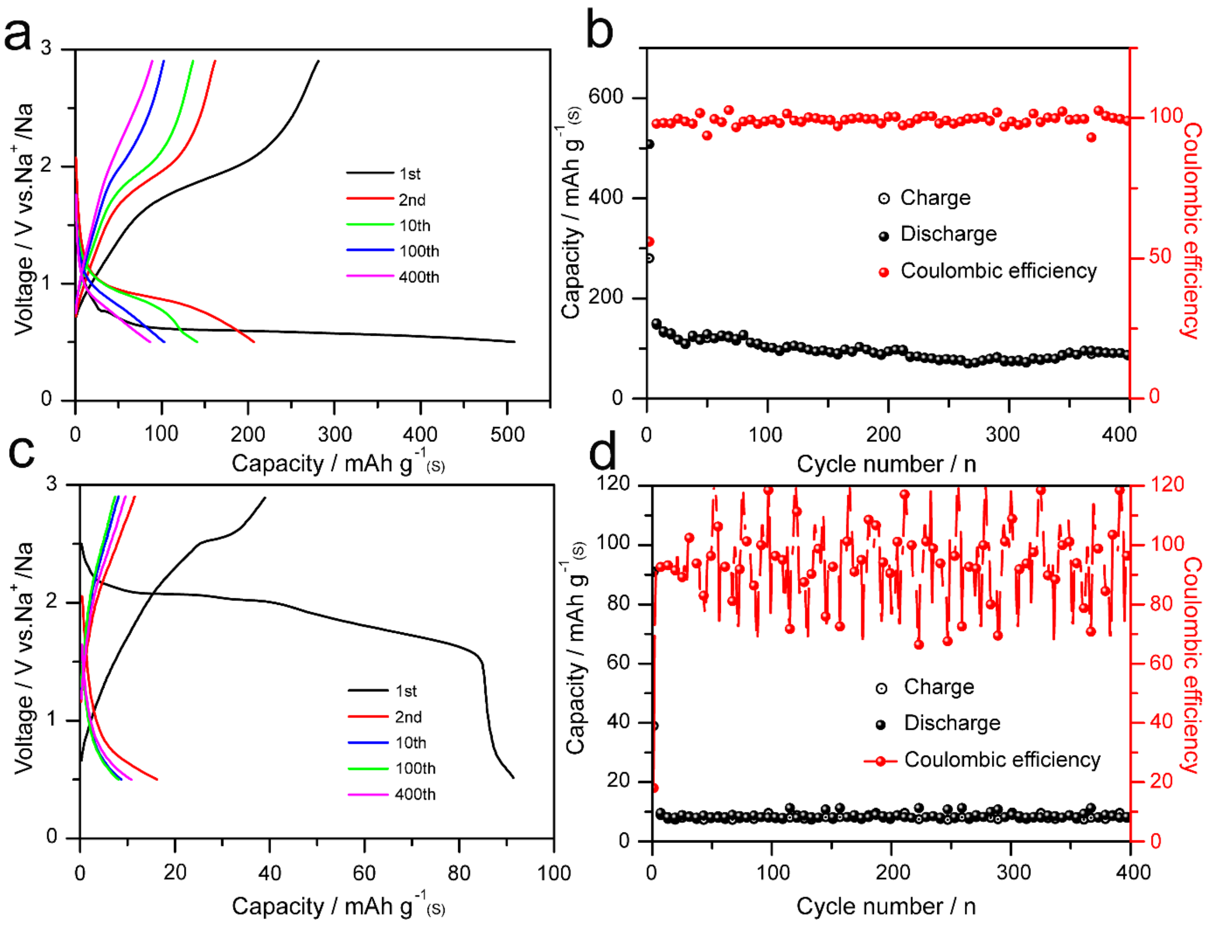

Figure S16. (a, c) Discharge/charge curves of the CC-40S and CAC-40S electrodes at 0.1 C. (b, d) Cycle performance of the CC-40S and CAC-40S electrodes at 0.1 C for 400 cycles.

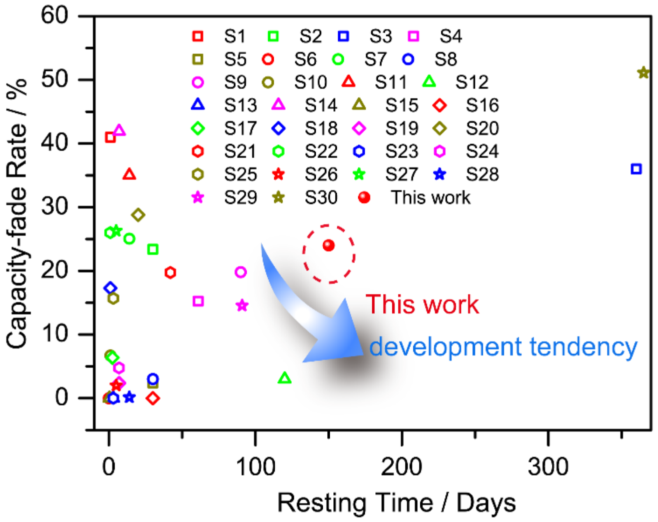

Figure S17. Static electrochemical analysis: time-dependent capacity-fade rate as a function of resting time. The references S1 – S30 refer to the research articles involving the development of low self-discharge.

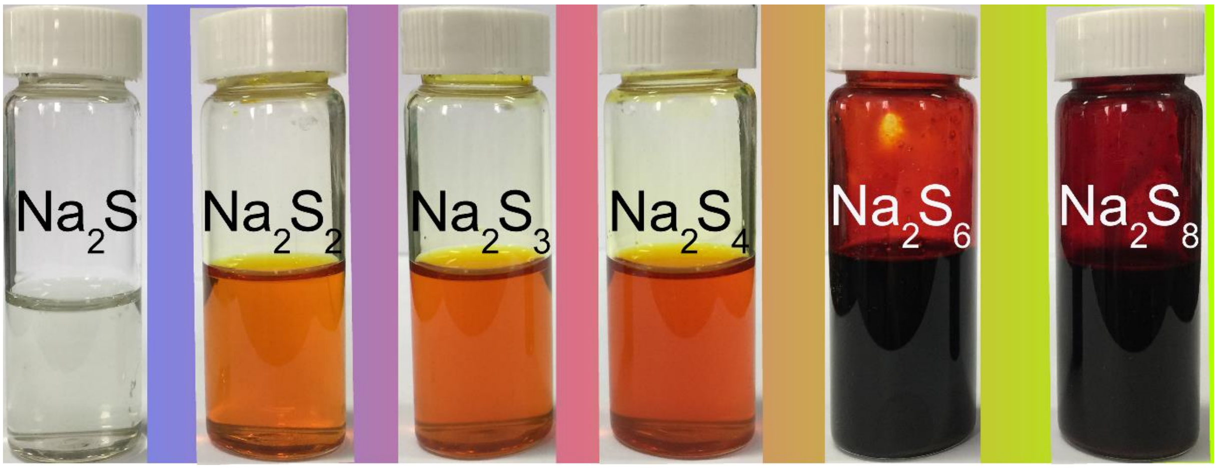

Figure S18. The photo images of Na_2S_X solutions.

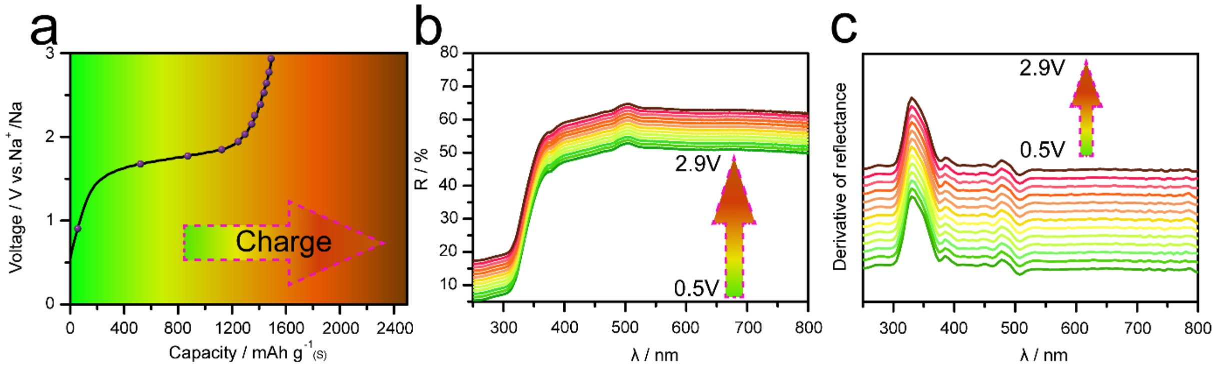

Figure S19. (a) Charge profile of the ACC-40S electrode at 0.1 C. (b) In situ UV/vis spectra and (c) homologous first-order derivative curves of the ACC-40S electrode.

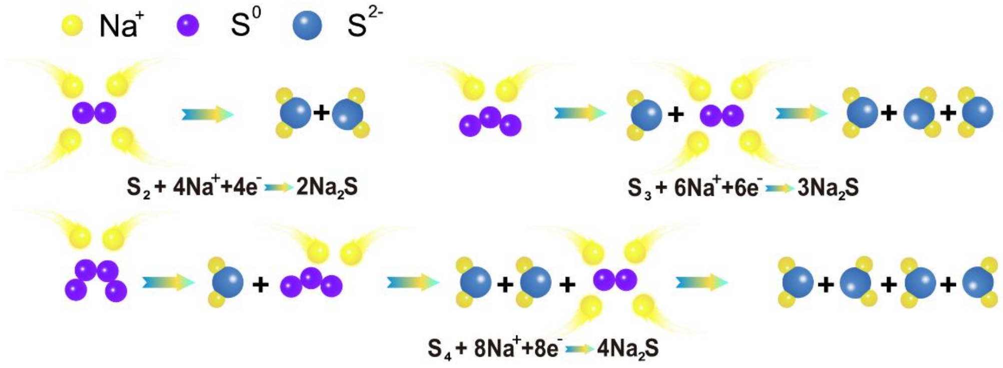

Figure S20. Schematic illustration of the reaction mechanism for converting S_2, S_3, and S_4 into Na_2S.

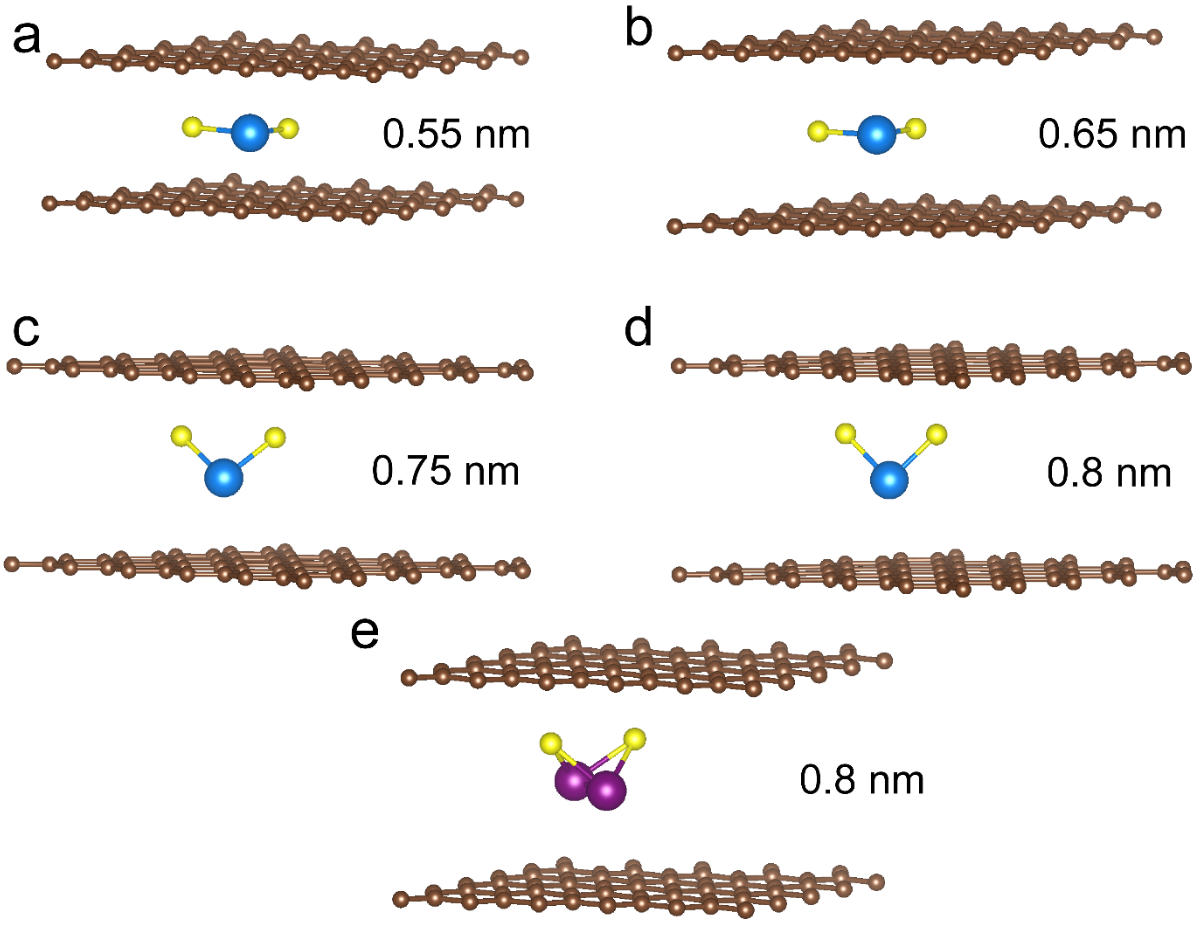

Figure S21. Optimized molecule structures of Na_2S and Na_2S_2 confined into the interlayers of the bilayer graphene with different interlayer spacings.

Table S1. To help visualize what kind of S-molecules could exist in the narrow slit micropores, we calculated the adsorption energies of S_2, S_3, S_4, S_5 and S_8 in slits from 5 to 7 Å. The adsorption energy is defined as $E_{ad} = E_{tot} - E_{bilayer} - E_{S\text{-molecule}}$, where E_{tot} is the total energy of the system, $E_{bilayer}$ denotes total energy of bilayer graphene, and $E_{S\text{-molecule}}$ is the free energy of sulfur molecule. The negative energy means insertion of S-molecule is an exothermal reaction.

Interlayer spacing	S_2	S_3	S_4	S_5	S_8
5.0 Å	2.34	3.69	5.09	5.15	8.14
5.5 Å	-0.06	0.48	1.54	1.77	3.57
6.0 Å	-0.91	-0.91	-0.28	1.63	3.64
7.0 Å	-1.16	-1.34	-1.28	-1.07	-1.24

Table S2 The adsorption energies of Na_2S on pristine graphene (p-G) and oxygen (O-G), hydroxyl (OH-G), nitrogen (N-G and 3N-G) functionalized graphene by using PBE+D3 method. The adsorption energy is defined as $E_{ad} = E_{tot} - E_{sub} – E_{Na2S}$, where E_{tot} is the total energy of the system, E_{sub} denotes total energy of pristine graphene or functionalize graphene, and E_{Na2S} is the free energy of isolated Na_2S molecular.

Na_2S	p-G	O-G	OH-G	N-G	3N-G
E_{ad}	-0.81	-1.54	-2.78	-.077	-2.17

References

[S1] Y. V. Mikhaylik, J. R. Akridge, *J. Electrochem. Soc.* **2004,** *151,* A1969−A1976.

[S2] H. S. Ryu, H. J. Ahn, K. W. Kim, J. H. Ahn, J. Y. Lee, E. J. Cairns, *J. Power Sources* **2005,** *140,* 365−369.

[S3] H. S. Ryu, H. J. Ahn, K. W. Kim, J. H. Ahn, K. K. Cho, T. H. Nam, *Electrochim. Acta* **2006,** *52,* 1563−1566.

[S4] S.-H. Chung,; A. Manthiram, *Electrochim. Acta* **2013,** *107,* 569−576.

[S5] L. Wang, X. He, J. Li, J. Gao, M. Fang, G. Tian, J. Wang, S. Fan, *J. Power Sources* **2013,** *239,* 623−627.

[S6] H. Chen, W. Dong, J. Ge, C. Wang, X. Wu, W. Lu, L. Chen, *Sci. Rep.* **2013,** *3,* 1910.

[S7] M. L. Gordin, F. Dai, S. Chen, T. Xu, J. Song, D. Tang, N. Azimi, Z. Zhang, D. Wang, *ACS Appl. Mater. Interfaces* **2014,** *6,* 8006−8010.

[S8] M. Kazazi, M. R. Vaezi, A. Kazemzadeh, *Ionics* **2014,** *20,* 1291−1300.

[S9] S.-H. Chung, A. Manthiram, *Adv. Funct. Mater.* **2014,** *24,* 5299−5306.

[S10] J.-Q. Huang, T.-Z. Zhuang, Q. Zhang, H.-J. Peng, C.-M. Chen, F. Wei, *ACS Nano* **2015,** *9,* 3002−3011.

[S11] F. Jeschull, D. Brandell, K. Edström, M. J. Lacey, *Chem. Commun.* **2015,** *51,* 17100−17103.

[S12] W.-T. Xu, H.-J. Peng, J.-Q. Huang, C.-Z. Zhao, X.-B. Cheng, Q. Zhang, *ChemSusChem* **2015,** *8,* 2892−2901.

[S13] L. Wang, Y. Wang, Y. Xia, *Energy Environ. Sci.* **2015,** *8,* 1551−1558.

[S14] H. Lu, K. Zhang, Y. Yuan, F. Qin, Z. Zhang, Y. Lai, Y. Liu, *Electrochim. Acta* **2015,** *161,* 55−62.

[S15] N. Azimi, Z. Xue, N. D. Rago, C. Takoudis, M. L. Gordin, J. Song, D. Wang, Z. Zhang, *J. Electrochem. Soc.* **2015,** *162,* A64−A68.

[S16] H. Chen, C. Wang, C. Hu, J. Zhang, S. Gao, W. Lu, L. Chen, *J. Mater. Chem. A* **2015,** *3*, 1392−1395.

[S17] G. Ai, Y. Dai, Y. Ye, W. Mao, Z. Wang, H. Zhao, Y. Chen, J. Zhu, Y. Fu, V. Battaglia, J. Guo, V. Srinivasan, G. Liu, *Nano Energy* **2015,** *16,* 28−37.

[S18] C.-Y. Fan, P. Xiao, H.-H. Li, H.-F. Wang, L.-L. Zhang, H.-Z. Sun, X.-L. Wu, H.-M. Xie, J.-P. Zhang, *ACS Appl. Mater. Interfaces* **2015,** *7,* 27959−27967.

[S19] L. Wang, J. Liu, S. Haller, Y. Wang, Y. Xia, *Chem. Commun.* **2015,** *51,* 6996−6999.

[S20] H. Lu, Y. Yuan, K. Zhang, F. Qin, Y. Lai, Y. Liu, *J. Electrochem. Soc.* **2015,** *162,* A1460−A1465.

[S21] H. Wang, W. Zhang, H. Liu, Z. Guo, *Angew. Chem. Int. Ed.* **2016,** *55,* 3992−3996.

[S22] J. Zhu, C. Chen, Y. Lu, J. Zang, M. Jiang, D. Kim, X. Zhang, *Carbon* **2016,** *101,* 272−280.

[S23] Q. Wang, Z. Wen, J. Jin, J. Guo, X. Huang, J. Yang, C. Chen, *Chem. Commun.* **2016,** *52,* 1637−1640.

[S24] L. Wang, J. Liu, S. Yuan, Y. Wang, Y. Xia, *Energy Environ. Sci.* **2016,** *9,* 224−231.

[S25] Q. Wang, Z. Wen, J. Yang, J. Jin, X. Huang, X. Wu, J. Han, *J. Power Sources* **2016,** *306,* 347−353.

[S26] T. Takahashi, M. Yamagata, M. Ishikawa, *Prog. Nat. Sci. – Mater.* **2015,** *25,* 612−621.

[S27] A. Ghosh, S. Shukla, G. S. Khosla, B. Lochab, S. Mitra, *Sci Rep.* **2016,** *6,* 25207.

[S28] H. Zhong, C. Wang, Z. Xu, F. Ding, X. Liu, *Sci Rep.* **2016,** *6,* 25484.

[S29] S.-H. Chung, C.-H. Chang, A. Manthiram, *Energy Environ. Sci.* **2016,** *9,* 3188−3200.

[S30] S.-H. Chung, P. Han, A. Manthiram, *ACS Appl. Mater. Interfaces* **2017,** *9,* 20318−20323.
